# Evaluation of IL-6 and High Sensitive C Reactive Protein Value in CSF and Serum Children Suspected Meningitis Referred to Pediatric Emergency Room

**DOI:** 10.5812/ircmj.4726

**Published:** 2012-12-06

**Authors:** Abdolkarim Hamedi, Hosain Ayatollahi, Alireza Ataee Nakhaee

**Affiliations:** 1 Mashhad University of Medical Science, Imam Reza Hospital, Mashhad, IR Iran; 2 Mashhad University of Medical Science, Ghaem Hospital, Mashhad, IR Iran

**Keywords:** Interleukin-6, Meningitis, Child

## Abstract

**Background:**

Acute bacterial meningitis which is a pediatric emergency with high mortality and morbidity must be diagnosed and treat promptly. Often diagnosis of bacterial meningitis from viral meningitis is difficult after some days. Determination of some inflammatory mediators’ example IL-6 and HS-CRP were useful in differential diagnosis of bacterial and viral meningitis.

**Objectives:**

This study attempted to Determining HS-CRP and IL6 in serum and CSF in children suspected meningitis and Comparing value HS-CRP and IL6 in bacterial/viral meningitis.

**Patients and Methods:**

Of children that hospitalized in pediatric emergency ward (Ghaem Hospital Mashhad university of medical science, in duration 01 Dec 2010-01 Dec 2011) and for they performed lumbar puncture, 1cc serum and CSF of they were taken to laboratory and have measured IL-6 with Elisa method and HS-CRP with immunoturbidometry method, patients were followed up and finally we compared levels of this two mediators.

**Results:**

Finally, this study performed on 81 children and infants. From 81 cases, 27 cases (33.3%) had bacterial meningitis 27 cases (33.3%) viral meningitis and 27 cases (33.3%) had normal CSF. IL-6 concentration in the CSF and serum were significantly raised in cases of bacterial meningitis (P = 0.001, P = 0.01) but HS-CRP concentration in the CSF and serum were not significantly raised in cases of bacterial meningitis (P = 0.46, P = 0.29). Mean IL-6 concentration in the CSF in bacterial meningitis was (180.74) and in viral meningitis was (39.08) .Mean HS-CRP in CSF in bacterial meningitis was (2.22) and viral meningitis was (1.29). Mean HS-CRP in serum in bacterial meningitis was (8.23) and viral meningitis was (6.36).

**Conclusions:**

The measurement of IL-6 in the CSF and serum in potentially a very useful diagnostic tool for differential diagnosis of bacterial and viral meningitis.

## 1. Background

Meningitis is the inflammation of leptomeninage (1). Acute bacterial meningitis which is a pediatric emergency with high mortality and morbidity must be diagnosed and treated promptly, to prevent severe complications. Differential diagnosis of bacterial from viral meningitis often is difficult after some days. There are some laboratory tests to differentiate these diseases: Acute stage of disease and acute phase reactant protein.In acute stage of disease, there is a change in some serum protein concentration that is acute phase response. Interleukin-6 (IL-6) activity and tumor necrosis factor-alpha concentrations in the cerebrospinal fluid (CSF) of patients with bacterial or viral, aseptic meningitis is useful for different type of infections (2). Increased levels of IL-6 and TNF were detected in the CSF of patients with bacterial meningitis (3). Acute phase reactant protein like serum procalcitonin is useful as a screening test for bacterial meningitis (4). Liver is the main organ which produces acute phase protein. Pro-Inflammatory mediators such as TNF-α, IL-1, IL-6 are majority these proteins, which are secreted from macrophages. CRP and other acute phase proteins as complement also participate in this phase and serum CRP 1000 times increased in the acute phase (5). Acute phase response also includes the increase in ACTH and Hydrocortisone and acute phase proteins and WBC count. CRP is 5 chain polypeptide-proteins.

## 2. Objectives

The measurement of IL6 and HS-CRP in serum and CSFof children suspected meningitis and compared bacterial and viral meningitis. Serum CRP is higher in bacterial than viral meningitis, and CRP of CSF is potentially useful diagnostic tool for diagnosis of bacterial meningitis. Therefore we measured these two mediators (IL6 and HS-CRP) in CSF and serum of children to differentiate bacterial from viral meningitis to prevent severe complications.

## 3. Patients and Methods

We preformed lumbar puncture in all children suspected meningitis. One (1cc) CSF was frozen in -20˚ and 1cc serum was santerifiuged in Lab, and then measured IL6 with Elisa and HS-CRP with Immunoturbidometry methods. Patients' designations were filled in a questionnaire to follow up them and then we compared these mediators in aseptic, bacterial and normal CSF. The gold standard of diagnosis bacterial and viral meningitis is culture and CSF analysis .Inclusion criteria: All children that have punctured spinal fluid, and exclusion criteria: All children who had not complete their CSF profile and final diagnosis.This information was analyzed by a computer and SPSS method. In this research, we described data, by using one – way ANOVA, Mann – Whitney test.

## 4. Results

Finally, this study performed on 81 cases of children, 27 cases had bacterial meningitis, 27 cases, viral meningitis, and 27 cases, normal CSF. Distribution of the patients’ age was between1-32 months (Mean 11.5months). Of 81 cases, 42 cases were male and 39 cases female. Mean serum IL6 in bacterial meningitis was (50.01) and in viral (10.64). Mean IL6 of CSF in bacterial meningitis was (180.74) and in viral (39.08). Mean serum HS-CRP in bacterial meningitis was (8.23) and in viral (6.36). Mean CSFHS-CRP in bacterial was (2.22) and in viral (1.29). Serum and CSF IL6 concentration were, significantly higher in bacterial than in viral meningitis, but serum and CSFHS-CRP in these two groups had no significant difference. The comparison of IL6 CSF concentration in these two groups (Bacterial and viral meningitis) of children, by use of Mann - Whitney test, show that difference is significant (P < 0.001). Moreover, mean IL6 CSF in children with aseptic meningitis is higher than in patient with normal CSF. Comparison of mean IL6 CSF concentration in these two groups of children by use of Mann - Whitney test, show that, difference is significant (P < 0.001); and mean IL6 CSF in children with bacterial meningitis is significant higher than in patient with normal CSF. CSF IL6 concentration is significantly higher in bacterial meningitis than in aseptic meningitis. (Tables 1, 2, 3, 4 and 5)

**Table 1:** Distribution of Patients on Age and Pattern of CSF

Age Pattern of CSF	Standard Deviation	Mean	Month, Min	Month, Max
**Normal**	12.25	14.09	1.5	32
**Aseptic**	8.33	9.30	1	27
**Bacterial**	12.47	13.3	<1	32

**Table 2:** Distribution of Serum Il6 Concentration in Two Groups of Children With Bacterial and Aseptic Meningitis

CSF Spec	Bacterial meningitis CSF, Mean ± SD	Aseptic Meningitis CSF, Mean ± SD	*P* value
**Mediators**			< 0.001
Serum IL6	50.01 ± 37.32	10.64 ± 10.7	

**Table 3:** Distribution of CSF IL6 Level in Two Groups of Children With Bacterial and Aseptic Meningitis

CSF Spec	Aseptic meningitis, CSF, Mean ± SD	Bacterial meningitis, CSF	*P* value
**Mediators**			< 0.01
HS-CRP in CSF	39.08 ± 35.6	180.74 ± 121.05	*** ***

Abbreviation: HS-CRP, high sensitive C - reactive protein

**Table 4:** Comparison of HS-CRP,CSF Frequency in Two Groups of Children with Bacterial Meningitis and Aseptic Meningitis

CSF Spec	Aseptic Meningitis CSF, Mean ± SD	Bacterial meningitis CSF, Mean ± SD	*P*value
**Mediators**			< 0.46
HS-CRPa, CSF	1.29 ± 1.18	2.22 ± 3.11	*** ***

Abbreviation: HS-CRP, high sensitive C - reactive protein

**Table 5:** Serum HSCRP Frequency Comparison in Two Groups of Children with Bacterial and AsepticMeningitis.

CSF Spec	Aseptic Meningitis CSF, Mean ± SD	Normal CSF, Mean ± SD	*P* value
**Mediators**			< 0/29
Serum HS-CRP	6.36 ± 3.96	8.23 ± 3.78	** **

Abbreviation: HS-CRP, high sensitive C - reactive protein

## 5. Discussion

Meningitis is one of the most emergency diseases in pediatric medicine with high mortality and morbidity, which must be diagnosed and treated promptly. There are some inflammatory mediators in CSF or serum that help to differential diagnosis of bacterial and viral meningitis such as IL6, CRP, Pre- calcitonin, TNF, IL8. In this study, we measured two inflammatory mediators, IL6 and HS-CRP, in CSF and serum of children who were admitted to pediatric emergency room and finally the level of these two mediators in three groups of patients were compared: Bacterial meningitis, viral meningitis, and normal CSF patients. In our study, mean HS-CRP CSF in bacterial meningitis was 2.22, in aseptic, 1.29 and in normal CSF, 0.68.Therefore HS-CRP CSF is not very useful for differentiating bacterial from aseptic meningitis, but can be useful for differentiating bacterial meningitis from normal CSF. We also measured serum HS-CRP in patients. According to our study, serum HS-CRP is not useful for differentiating bacterial meningitis from aseptic meningitis, but is useful diagnostic tool for differentiating bacterial meningitis from normal CSF. Measurement of CSF and serum IL6 is potentially useful for differentiating normal CSF patients from bacterial meningitis. IL6 level in CSF and serum is useful diagnostic tool for differential diagnosis of bacterial and viral meningitis. Celik et al. in 2007, found no standard method for rule out of bacterial meningitis in patients whom CSF cytology is according to bacterial meningitis, but CSFculture and gram stain is negative. Therefore, some factors in CSF need to be determined. The aims of this study, is retrospective comparison of WBC count, CRP, ESR, WBC and neutrophil count of CSF were independent variable that in regression model, these have 45% positive predictive value in bacterial meningitis and 93.2% PPV in viral (6). In another study, Dr Taskin et al. in 2004, determined pre-calcitonin and another cytokines level in CSF of children to differentiate bacterial meningitis from viral meningitis (7). Gendral et al. (1998), compared serum pre – calcitonin and CSF CRP and IL6, to differentiate these diseases, pre – calcitonin level was measured in 23 children with bacterial meningitis and 51 patients with viral meningitis (age of patients, from 3 mo-14yr) (8). Mean serum CRP was143/3 mg/l (28-351) in bacterial meningitis and 13.9 (1-48) in viral. Mean CSF CRP was 2.2 (0.4-4.74) in bacterial meningitis and 0.57 (0.12-2.72) in viral and WBC in CSF was (240-17500) in bacterial meningitis and (20-3200) in viral, that not sufficient useful to differentiate viral meningitis from bacterial. 24 patients From 54 cases, 24 patients had viral meningitis which were treated by antibiotic. IL6 level in admission of 1.8 patients with bacterial meningitis was less than 100pg and in 7.19 of patients with viral meningitis was greater than 100pg. pre – calcitonin level in all groups of patients was useful in differential diagnosis of meningitis because mean pre – calcitonin concentration was 61mg (4.8-33.5) in bacterial meningitis and 0.33mg (0-1.7), in viral, after antibiotic therapy, pre-calcitonin level decreased to low level than cannot be detected so pre – calcitonin level is sensitive and specific mediator for differential diagnosis of viral and bacterial meningitis. Skarman et al. (1994), measured CRP, TNF, IL6 and CBC diff in blood of 24 patients with manifestation of meningitis and in control group, CRP level did not increase in patient with viral meningitis compared to control group. CRP had 87% sensitivity in bacterial meningitis diagnosis. TNF of CSF increased significantly in bacterial meningitis, (P < 0.001)in viral meningitis, it increased various (9). In one study in India, in 2001, CRP was measured in serum and CSF of 47 patients as a factor of rapid diagnosis of bacterial meningitis (10). The increase of CRP in CSF and blood is indicative of invasive infection. 46.66% of these patients had positive CRP in CSF but 88% had positive HS-CRP. Statically, serum CRP level was greater than 18mg/dl and CSF CRP greater than 8mg/dl that is valuable. In one study in USA (2007) considered that pre – calcitonin level is higher in bacterial meningitis than in viral meningitis (11). In 2005, CRP, IL7, IL18 and B1 were measured in neuroburiliasis patients, were efficient in diagnosis of lyme meningitis (12). In Japan (2001), HS-CRP was positive in all patients who had bacterial meningitis (HS-CRP>100), but only was positive in 10% viral meningitis (13). In Gambia and Etupia (2004), CRP level in 466 infants was less than 3mo age which was correlated with positive culture. CRP>10mg/dl had 55% sensitivity and 82% specificity but CRP>40 had 55% sensitivity and 82% specificity (14). Peltola et al. (1985) described that serum CRP level in all patients with bacterial meningitis was greater than upper limit of normal, (>19mg/dl) and mean level was 195mg/dl (155-375mg/dl) (15). In epidemiologic study, measured IL6 of CSF is important and useful in diagnosis of meningitis (16).In 12 patients with viral meningitis, serum CRP level was normal. As a result, serum CRP in bacterial meningitis is always detectable. In one study in 1992, IgE, and IL6 was measured in CSF and serum of patients with bacterial and viral meningitis. As a result, IL6 and IgE level CSF were important factors for evaluation of patients and prognosis of disease (16). Gary in 1986 measured CRP in CSF of 145 patients with meningitis. CRP level that obtained, in 49 patients with bacterial meningitis and positive culture was (0-51000ng/ml). In 33 patients with aseptic meningitis, the level was lower (0-438ng/ml) (mean = 17ng/ml). Pleocytosis of CSF with greater than 10 WBC/ml and CRP>100ng/ml had 95% positive diagnostic value for bacterial meningitis; nevertheless, few patients with no or little WBC in CSF had low CRP in admission (17). Therefore, CRP in CSF potentially is a useful diagnostic tool, but some patients have not enough rapid inflammatory response. In acute bacterial meningitis, CRP and pre – calcitonin rise in serum and CSF (18). These are useful in differential diagnosis of Bacterial and viral meningitis. In Jamil report, IL-la had the highest sensitivity and negative predictive value in meningitis and, IL-6 had the highest specificity and positive predictive value (19). In infants with suspected neonatal sepsis, two CRP samples that are < 10 mg/l are useful in excluding sepsis (20). In malignancy, increased serum levels of IL-6 correlates with the extent of tumor invasion, and metastasis (21). Finally, inflammatory mediators’ example of IL-6 and HS-CRP are useful in differential diagnosis of bacterial and viral meningitis.

The measurement of IL-6 in the CSF and serum is potentially a very useful diagnostic tool for differential diagnosis of bacterial and viral meningitis. CRP in CSF potentially is a useful diagnostic tool, but some patients have not enough rapid inflammatory response. HS-CRP in CSF is better for differential diagnosis of bacterial and viral meningitis.
